# The Effects of Different Types of Sleep Disorder on Colorectal Cancer: A Nationwide Population-Based Cohort Study

**DOI:** 10.3390/cancers15194728

**Published:** 2023-09-26

**Authors:** Po-Lin Chiang, Wen-Rui Hao, Hong-Jye Hong, Chun-Chao Chen, Chun-Chih Chiu, Yu-Ann Fang, Tsung-Lin Yang, Yu-Hsin Lai, Ming-Yao Chen, Min-Huei Hsu, Kuan-Rau Chiou, Kuan-Jie Lin, Tsung-Yeh Yang, Hsin Hsiu, Ju-Chi Liu

**Affiliations:** 1 Division of Cardiology, Department of Internal Medicine, Shuang Ho Hospital, Taipei Medical University, New Taipei City 23561, Taiwan15535@s.tmu.edu.tw (T.-Y.Y.); 2 Department of General Medicine, Taipei Veterans General Hospital, Taipei 11217, Taiwan; 3 Taipei Heart Institute, Taipei Medical University, Taipei 11031, Taiwan21514@s.tmu.edu.tw (K.-J.L.); 4 Division of Cardiology, Department of Internal Medicine, School of Medicine, College of Medicine, Taipei Medical University, Taipei 11031, Taiwan; 5 School of Chinese Medicine, College of Chinese Medicine, China Medical University, Taichung City 404333, Taiwan; 6 Graduate Institute of Medical Sciences, College of Medicine, Taipei Medical University, Taipei 11031, Taiwan; 7 Division of Cardiology, Department of Internal Medicine and Cardiovascular Research Center, Taipei Medical University Hospital, Taipei 11031, Taiwan; 8 Division of Gastroenterology and Hepatology, Department of Internal Medicine, School of Medicine, College of Medicine, Taipei Medical University, Taipei 11031, Taiwan; 09698@s.tmu.edu.tw (Y.-H.L.);; 9 TMU Research Center for Digestive Medicine, Taipei Medical University, Taipei 11031, Taiwan; 10 Division of Gastroenterology and Hepatology, Department of Internal Medicine, Shuang Ho Hospital, New Taipei City 23561, Taiwan; 11 Graduate Institute of Data Science, College of Management, Taipei Medical University, Taipei 11031, Taiwan; 12 Department of Neurosurgery, Shuang Ho Hospital, Taipei Medical University, New Taipei City 23561, Taiwan; 13 Division of Cardiovascular Surgery, Department of Surgery, Shuang Ho Hospital, Taipei Medical University, New Taipei City 23561, Taiwan; 14 Graduate Institute of Biomedical Engineering, National Taiwan University of Science and Technology, No. 43, Section 4, Keelung Road, Taipei 10607, Taiwan

**Keywords:** sleep disorder, sleep apnea, insomnia, colorectal cancer, malignancy

## Abstract

**Simple Summary:**

In this study, we used Taiwan’s National Health Insurance Research Database to investigate the potential association between sleep disorder (SD) and colorectal cancer (CRC) after matching 177,707 patients with SD and without a history of CRC with 177,707 non-SD and non-CRC Taiwanese citizens without SD and without CRC based on age and gender. The results demonstrated a significantly higher likelihood of developing CRC in SD patients compared to the control group. Subgroup analysis revealed that among the four different types of SD, the insomnia group exhibited a significantly elevated risk of CRC. It should be noted that individuals simultaneously affected with sleep apnea and insomnia had a significantly higher risk of developing CRC than those with either condition alone. These research findings suggest a potentially higher risk of CRC in Taiwanese SD patients, underscoring the need to explore the relationship between SD and CRC risk.

**Abstract:**

The impact of sleep disorders (SDs), particularly sleep apnea (SA), on the development of colorectal cancer (CRC) has been the subject of significant research. However, the potential contribution of other SDs to the incidence of CRC remains unexplored. The objective of this study was to examine the effects of SDs on the risk of developing CRC. This study assessed CRC risk among individuals diagnosed with SDs compared with age- and sex-matched unaffected individuals. A longitudinal, nationwide, population-based cohort study was conducted using data from the Taiwan National Health Insurance Research Database (NHIRD) encompassing 177,707 individuals diagnosed with SDs and 177,707 matched controls. Cox proportional hazard regression analysis was used to determine the relative increased risk of CRC in individuals with SDs and specific subgroups of SDs. The CRC incidences were 1.32-fold higher (95% CI 1.23–1.42) in the overall SD cohort, 1.17-fold higher (95% CI 0.82–1.68) in the SA cohort, 1.42-fold higher (95% CI 1.31–1.55) in the insomnia cohort, 1.27-fold higher (95% CI 1.17–1.38) in the sleep disturbance cohort, and 1.00-fold higher (95% CI 0.77–1.29) in the other SD cohort, after adjusting for age, sex, and comorbidities.

## 1. Introduction

Colorectal cancer (CRC) is the third most commonly diagnosed and the fourth most deadly cancer worldwide [1,2]. Many risk factors are known to play a major role in the development of CRC, including age, genetics, environment, a Western lifestyle, cigarette smoking, BMI/obesity, alcohol consumption, and certain dietary habits [3,4]. Even so, the incidence and mortality of CRC have been decreasing in recent years due to effective screening measures such as fecal occult blood testing and colonoscopies [3,4,5]. Despite that, CRC is responsible for approximately 900,000 deaths each year [6]. As a result, it is critical to explore risk factors and associations between other diseases and CRC.

Sleep is a complicated physiological process that affects both the autonomic nervous system (ANS) and cellular inflammatory signaling [7,8]. Sleep disorders (SDs) are categorized into four types, including sleep apnea (SA), insomnia, sleep disturbance, and other SDs. Poor or insufficient sleep has been found to be associated with a wide variety of diseases, including cardiovascular diseases, hypertension, dementia, and depression [9,10,11,12]. Several studies have focused on the correlation between SDs and the incidence of cancers and found that short or long sleep duration has an impact on the prevalence rate of CRC [13].

In 2019, Lin et al. used data from the Taiwan National Health Insurance Research Database (NHIRD) to investigate the association between SDs and CRC [14]. The researchers defined two cohorts—individuals with CRC and those without CRC—and discussed the effect of depression on CRC prevalence [14]. However, Lin et al. did not conduct subgroup analyses of SDs, and they did not take into account the fact that some patients were diagnosed with more than one type of SD. We postulate that different types of SDs might differentially affect the prevalence of CRC; therefore, we utilized data from the Taiwan NHIRD to conduct a longitudinal retrospective cohort study to verify the findings of Lin et al. and better understand the impact of SDs on CRC.

## 2. Materials and Methods

The Taiwan NHIRD includes records of medical claims for inpatient, outpatient, and ambulatory care. The NHIRD is a comprehensive dataset covering approximately 99% of Taiwan’s over 23 million residents and has been used in many previous studies [15,16,17,18]. The National Health Insurance (NHI) program has been in effect since 1995 and provides coverage to nearly all Taiwanese residents. Diagnoses are coded by physician specialists according to the International Classification of Diseases, Ninth Revision, Clinical Modification (ICD-9-CM). Several studies have confirmed the accuracy and validity of diagnoses in the NHIRD [19,20]. Anonymity is guaranteed when data are sent to the National Health Research Institutes (NHRI) for database construction, and further deidentification measures are enacted before the data are released to researchers. This study was approved by the Joint Institutional Review Board of Taipei Medical University (approval no. N201804043).

### 2.1. Participants

**Figure 1:**
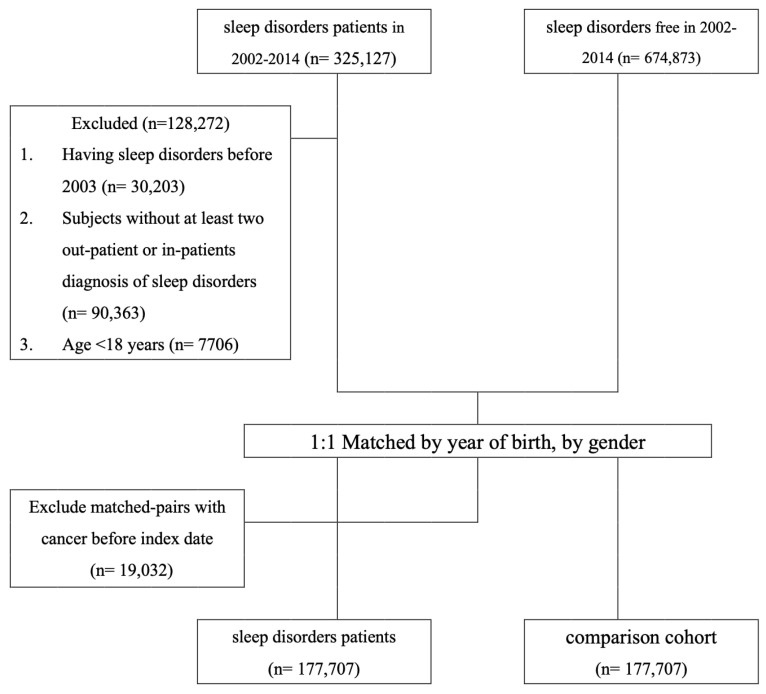
Data selection process.

Our study cohort consisted of all patients diagnosed with SDs between 1 January 2002 and 31 December 2014. We used a 1-year (2002) washout period to warrant that no patients in the cohort had an SD prior to enrollment (n = 30,323; Figure 1). Patients who had fewer than two SD diagnoses as outpatients or one diagnosis as inpatients were excluded (n = 90,363; Figure 1), and patients younger than 18 years old were also excluded (n = 7706; Figure 1). The final group of patients to be analyzed was divided into four case cohorts: an SA cohort (ICD-9-CM codes 780.51, 780.53, and 780.57); an insomnia cohort (ICD-9-CM code 780.52); a sleep disturbance cohort (ICD-9-CM codes 780.5, excluding 780.51, 780.53, 780.57); and others (ICD-9-CM codes 307.4, 780.54–780.56, and 780.58–780.59). Each patient with an SD was matched with a non-SD patient according to birth date and gender. Any participant in the matched pairs who had a history of CRC was excluded (n = 19,032; Figure 1). A total of 177,707 pairs were successfully matched. Both cohorts had the same index date, which was defined as the first occurrence of a diagnosis of SD for follow-up. All of the cohorts were followed until an initial diagnosis of CRC (ICD-9-CM code 153.X-154.X) was made, the patient was lost to follow-up, death, or withdrawal from the NHI, or the study duration elapsed on 31 December 2014.

### 2.2. Potential Confounders

The following covariates were investigated and compared between the two cohorts to establish the baseline characteristics of each individual: Charlson Comorbidity Index (CCI) (categorized into four groups: 0; 1; 2; and ≥3) [21]; diabetes (ICD-9-CM code 250.X); hypertension (ICD-9-CM codes 401.X-405.X); dyslipidemia (ICD-9-CM code 272.X); atrial fibrillation (ICD-9-CM code 427.31); and prescriptions for medications that included aspirin, statin, renin-angiotensin-aldosterone (RAA) system inhibitor, and metformin. The cohort was also classified according to sociodemographic characteristics: age (categorized into three groups: 18–44; 45–64; and ≥65 years old); gender (male, female); level of urbanization (urban, suburban, and rural area); and monthly income (0, 1–21,000, 21,000–33,300, and ≥33,301 New Taiwan Dollars (NT$), which corresponds to 0, 0.03–700, 700–1100, and ≥1100 USD).

### 2.3. Statistical Analysis

Chi-squared analyses were used to compare the SD and comparison cohort concerning age (18–44 years, 45–64 years, and ≥65 years), gender, CCI, comorbidities, medications, level of urbanization, and monthly income. A *t*-test was used to identify differences between the SD and comparison cohorts for the continuous variables listed in Table 1. Hazard ratios (HRs) and 95% confidence intervals (95% CIs) for the association between SD and the risk of CRC were examined using Cox proportional hazards regression analysis in Table 2. The relative risks of CRC for the SA, insomnia, sleep disturbance, other SDs, and comparison cohorts are listed in Table 3. Additionally, we compared the incidence rates of CRC between the SA-only group, the insomnia-only group, and the group diagnosed with both SA and insomnia using stratified analysis across different age and gender groups; the findings are outlined in Table 4. The Kaplan–Meier method was used to calculate the cumulative event rates of CRC, and the log-rank test was used to evaluate statistical significance. All of the analyses were conducted using SAS statistical software (Version 9.4 for Windows; SAS Institute Inc., Cary, NC, USA). Statistical significance was set at *p* < 0.05 for a two-tailed test.

## 3. Results

### 3.1. Baseline Characteristics of the SD and Non-SD Groups

From 2002 through 2014, a total of 177,707 patients were newly diagnosed with SDs, including 4018 patients with SA, 66,648 patients with insomnia, 99,789 patients with sleep disturbance, and 7252 patients with other SDs. The mean age of the SD cohort was 45.86 years, which was similar to that of the control cohort. Over 85% of the participants were under 65 years of age. Patients in the SD cohort were more likely to have higher CCI scores, as well as comorbidities such as diabetes, hypertension, dyslipidemia, and atrial fibrillation; they were also more likely to use aspirin, statins, RAA, or metformin compared with the control cohort (*p* < 0.001) (Table 1).

### 3.2. Sex- and Age-Stratified Analyses of the SD and Non-SD Groups

The overall incidence of CRC in the SD cohort was significantly higher than in the control cohort (162.1 vs. 116.1 per 100,000 person-years). The adjusted HR was 1.32 (95% CI 1.23–1.42), representing a 1.4-fold increased risk (Table 2). Age-stratified analyses revealed that individuals in the SD cohort had a higher likelihood of developing CRC compared with individuals in the control cohort across all three age groups; the adjusted HRs and 95% CIs were 1.66 (95% CI 1.39–1.98), 1.26 (95% CI 1.13–1.40), and 1.27 (95% CI 1.14–1.42). The adjusted HRs were 1.38 (95% CI 1.25–1.51) for women and 1.27 (95% CI 1.14–1.40) for men, compared with their non-SD counterparts. Men had a higher incidence of CRC than women, with an incidence rate of 149.9 (95% CI 138.7–161.2) and 197.3 (95% CI 148.3–210.4) per 105 person-years for non-SD and SD individuals, respectively. The corresponding incidence rates for women were 96.1 (95% CI 89.1–103.0) and 141.4 (95% CI 133.0–149.9) per 105 person-years for non-SD and SD individuals, respectively.

### 3.3. Sensitivity Analyses

Among the four subgroups of individuals with SDs, the adjusted HRs and 95% CIs for CRC were 1.17 (95% CI 0.82–1.68), 1.42 (95% CI 1.31–1.55), 1.27 (95% CI 1.17–1.38), and 1.00 (95% CI 0.77–1.29) compared with the control cohort (Table 3). The individuals in the insomnia subgroup had a significantly higher risk of developing CRC across all age groups; the adjusted HRs were 1.93 (95% CI 1.54–2.43), 1.41 (95% CI 1.24–1.60), and 1.30 (95% CI 1.14–1.48). We conducted a series of additional analyses on the insomnia subgroup to account for the effects of medication use; we found that, compared with the control cohort, patients with insomnia had higher adjusted HRs (aspirin, 1.48 (95% CI 1.36–1.61), statin, 1.46 (95% CI 1.34–1.59), RAA, 1.48 (95% CI 1.36–1.61), and metformin, 1.43 (95% CI 1.31–1.55)). In addition, we performed additional analyses on the patients with SA only, insomnia only, those who had both SA and insomnia, and the control cohort. The adjusted HRs and 95% CIs for CRC for the aforementioned groups were 1.08 (95% CI 0.69–1.69), 1.45 (95% CI 1.31–1.61), and 1.66 (95% CI 1.21–2.29) (Table 4). In the subgroup analysis of patients with both SA and insomnia, stratified by different age groups, the subjects with both SA and insomnia had a higher risk of developing CRC compared with the control cohort. The adjusted HRs were 2.57 (95% CI 1.17–5.67), 1.57 (95% CI 1.00–2.49), and 1.51 (95% CI 0.86–2.63). However, significant differences were only found for the youngest patients.

### 3.4. Cumulative Incidence of CRC among Different Subgroups

**Figure 2:**
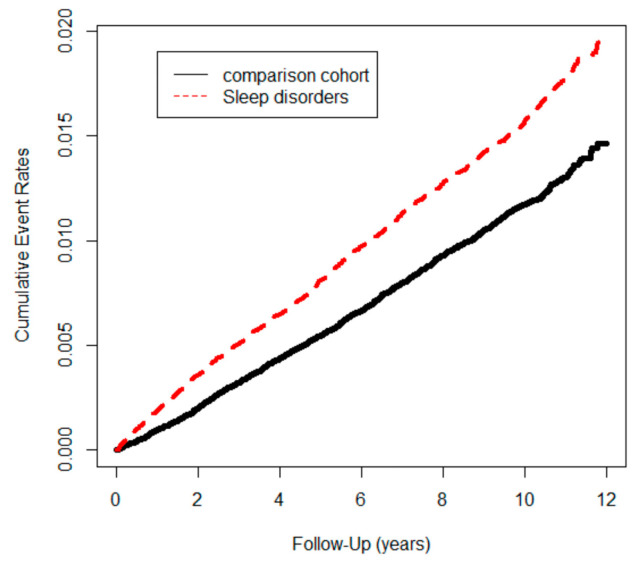
Colon cancer events in study cohort (n = 355,414) from 1 January 2001 to 31 December 2012 in Taiwan, stratified by sleep disorders and comparison cohort (log-rank test, χ^2^ = 92.4; df = 1; *p* < 0.001.).

**Figure 3:**
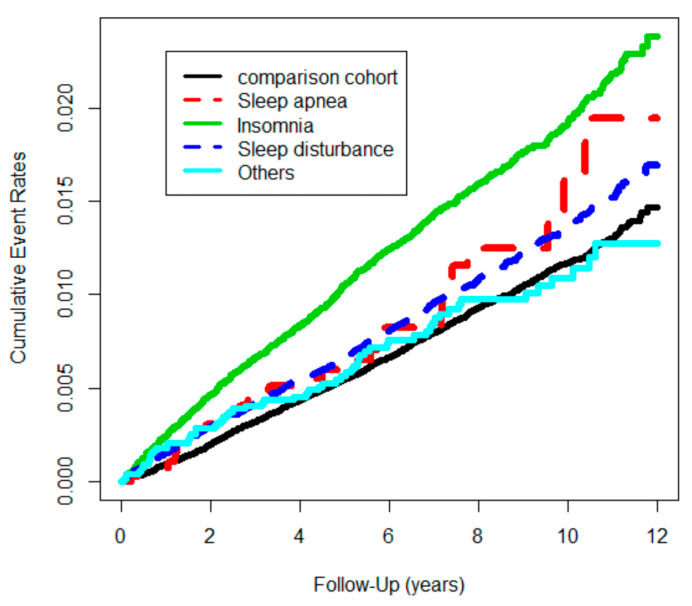
Colon cancer events in study cohort (n = 355,414) from 1 January 2001 to 31 December 2012 in Taiwan, stratified by sleep disorders and comparison cohort (log-rank test, χ^2^ = 182; df = 4; *p* < 0.001.).

The cumulative incidence of CRC was significantly higher in the SD cohort compared with the control cohort (log-rank test, χ^2^ = 92.4; df = 1; *p* < 0.001) until the end of the follow-up (Figure 2). Furthermore, the cumulative incidence of CRC was significantly higher in the insomnia cohort compared with other subgroups (log-rank test, χ^2^ = 182; df = 4; *p* < 0.001) until the end of the follow-up (Figure 3).

This longitudinal, large-scale, nationwide, population-based retrospective cohort study included 355,414 patients in Taiwan. The results can be summarized as follows: (1) SDs were associated with a significantly higher risk of CRC across all patient age and gender groups; (2) patients with insomnia had a higher likelihood of developing CRC; patients with SA and other SDs had a lower likelihood of developing CRC; (3) in the insomnia group and the subgroups with zero CCI, patients who did not have diabetes, dyslipidemia, or hypertension had a higher HR, a finding that suggests that the risk of CRC is independent of risk factors that have been traditionally perceived to be carcinogenic; (4) although SA appeared to be insignificantly linked to CRC, the risk of CRC in patients with both insomnia and SA was significantly higher (1.66-fold) than in patients without these conditions (SA 1.08-fold, insomnia 1.45-fold).

## 4. Discussion

To the best of our knowledge, this is the first population-based, longitudinal cohort study that has investigated the risk of CRC in association with SDs and the joint effect of SA and insomnia. Previous research showed that patients with colorectal adenoma had a higher prevalence of self-reported SA compared with a control group [22]. Cheng et al. and Brenner et al. both performed a meta-analysis and suggested that obstructive SA (OSA) may be associated with an increased incidence of overall cancers; however, the association with a specific type of cancer was not significant [23,24]. Brenner et al. reported that this trend was especially pronounced among patients younger than 45 with a high apnea–hypopnea index (AHI) [24]. Another large meta-analysis that followed up patients for a median duration of 7–11 years revealed that the incidence of CRC was 1.70-fold higher in an OSA cohort compared with the control cohort [25]. Similarly, Zhou et al. reported that both the AHI and oxygen desaturation index (ODI) were positively associated with levels of carcinoembryonic antigen (CEA), which is used for detecting CRC (particularly in individuals with a history of BMI/obesity, smoking, or inadequate glycemic management) [26]. Xiong et al. noted that ODI may be a useful prognostic factor for CRC patients [27]. In 2019, one population-based study that also used data from the Taiwan NHIRD indicated that the OSA cohort had a significantly increased risk (1.80-fold higher) of developing CRC compared with the comparison cohort; furthermore, increased visits for OSA were significantly correlated with a higher risk of CRC [28]. In our study, data from the SA group only revealed a slight increase in the risk of developing CRC; this phenomenon or bias may be due to the presence of undiagnosed SA patients in the comparison group. To minimize potential bias, we conducted an adjusted HR analysis that considered various factors (i.e., age, gender, comorbidity index, diabetes, dyslipidemia, hypertension, atrial fibrillation, medication use, level of urbanization, and monthly income). Moreover, an advantage of our study lies not only in its large sample size and longer follow-up period but also in the more extensive classification of SDs compared with previous investigations.

Research on the association between insomnia and CRC, as well as on sleep disturbance and CRC, is very limited; more investigations have focused on SA. A meta-analysis involving 578,809 subjects conducted by Shi et al. revealed a 24% increased risk of cancer in patients with insomnia compared with patients without insomnia; this trend was particularly noted in women [29]. Another meta-analysis of nearly 1.6 million insomnia patients noted that issues of difficulty falling asleep (DFA) and non-restorative sleep (NRS) were linked to increases in all-cause mortality and cardiovascular disease mortality in elderly patients [30]. Chen et al. conducted a prospective study of 392,252 individuals and found that a daily sleep duration of 7–8 h and no frequent episodes of insomnia reduced the risk of CRC by 9% and 14%, respectively; these authors emphasized the correlation between a healthy lifestyle and CRC [31]. In the year 2023, two studies explored the association between insomnia and CRC. Yoon et al. conducted a nationwide retrospective cohort study with nearly four million samples. Insomnia may either increase or decrease the risk of certain cancers, and this relationship can vary based on age, gender, and the type of cancer. [32] On the other hand, Wong et al. conducted a consecutive cohort study that included 18,302 patients receiving palliative care for CRC. They found a higher probability of clinically significant insomnia or any form of insomnia among patients with advanced CRC. [33] Our findings revealed that the risk of CRC in patients with both SA and insomnia was significantly higher (1.66-fold) than in patients without these conditions (SA 1.08-fold, insomnia 1.45-fold). Prior evidence has supported a bidirectional association between SA and insomnia known as comorbid insomnia and SA (COMISA) [34]. However, to date, there is still a lack of research specifically examining the relationship between COMISA and cancer; the literature has primarily focused on exploring the symptoms, evaluation, and treatment modalities of COMISA [34,35]. It is important that future studies clarify the direct or indirect effects of SA and insomnia on CRC.

### 4.1. Mechanisms behind the Association between the Risk of CRC and SDs

The pathophysiological mechanisms between SDs and CRC remain unclear. Currently, the two most studied aspects include circadian rhythm disruption and cellular hypoxia. Disruption of circadian rhythm and cellular hypoxia are fundamental components of tumor cell metastasis. These two processes regulate pathological alterations in tumor cell metabolism and can result in a loss of differentiation and the acquisition of an invasive phenotype. Furthermore, they stimulate pro-metastatic modifications in tumor stroma cells, including macrophages and endothelial cells, which act in tandem to promote the systemic spread of cancer [36,37].

The body’s circadian clock regulates a wide range of physiological processes, including cell proliferation, DNA damage repair, and apoptosis. Disruptions in circadian rhythmicity have been linked to alterations in these processes that can promote tumor growth and metastasis [38,39,40]. Moreover, circadian dysfunction has been linked to alterations in the gut microbiome that can contribute to the development of CRC [41]. Disruptions in circadian rhythm have been shown to alter the composition of the gut microbiome and promote inflammation, which can, in turn, contribute to tumor initiation and progression [42,43].

Hypoxia is a common feature of solid tumors, including CRC, due to the rapid growth of tumor cells that outstrips the ability of blood vessels to supply oxygen [44]. This process results in a metabolic shift in which tumor cells rely more on glycolysis instead of oxidative metabolism to produce energy (i.e., the Warburg effect) [45]. This metabolic shift not only allows tumor cells to survive in low-oxygen conditions but also promotes their growth and invasion into surrounding tissues [37]. Furthermore, hypoxia has been linked to the activation of several signaling pathways that contribute to the aggressive behavior of CRC cells. For instance, hypoxia-inducible factor 1 (HIF-1) is a vital transcription factor that governs the expression of various genes that are crucially involved in critical cellular processes such as angiogenesis, cell viability, and metastasis [46]. HIF-1 is stabilized under hypoxic conditions and promotes the expression of genes that enhance tumor cell survival and proliferation [47]. Additionally, hypoxia has been shown to induce epithelial-mesenchymal transition (EMT), a process by which cancer cells acquire invasive properties and become resistant to chemotherapy [48].

### 4.2. Limitations

This study is characterized by certain limitations, given that it is based on an electronic insurance database. First, given the potential for coding errors and upcoding in the NHIRD cohort research, we implemented a more rigorous diagnostic criterion to reduce potential coding biases: only individuals who had made at least two visits to an outpatient clinic or were admitted to the hospital for an SD were included in the study sample. Second, the lack of polysomnography outcomes in the NHIRD hindered differentiation between central and obstructive SA, which could have affected our results. However, this study still confirmed a correlational trend between the majority of SA cases and the incidence of CRC. Future studies should focus on the relationship between different types of SA and CRC. An additional limitation of this study is that it makes use of the ICD-9 coding system instead of ICD-10, which is currently widely used in Taiwan. However, the ICD-9 coding classification for SDs is generally similar to that of ICD-10 (for SA, ICD-9-CM 780.51, 780.53, and 780.57 can be converted to ICD-10-CM G47.30; for insomnia, ICD-9-CM 780.52 can be converted to ICD-10-CM G47.00). We do not expect that this limitation will have a significant impact on our results. Fourth, common risk factors of CRC were not adjusted as covariates due to the lack of information in the database, including BMI/obesity, tobacco use, alcohol consumption, low-fiber and high-fat diets, as well as lack of physical activity. Especially concerning BMI/obesity, several studies utilizing national databases have faced this limitation [14,28,49]. Therefore, we recommend that future research should consider the association between BMI/obesity and sleep disorders as well as CRC. Future studies that link these patients to another registry will be important for investigating these possible confounders. To ensure the veracity of these findings, subsequent studies should integrate thorough polysomnography and related diagnostic procedures.

## 5. Conclusions

We found a significant association between SDs and the incidence of CRC; insomnia exerted a stronger effect on CRC than other types of SDs. Additionally, we noted that individuals who have both insomnia and SA are at a higher risk for CRC than patients with either condition alone. The elevated risk of CRC among patients with insomnia may be independent of factors such as age, sex, comorbidities, and medication history.

## Figures and Tables

**Table 1:** Characteristic of the Sample Population.

	Whole Cohort (n = 355,414)		Sleep Disorders (n = 177,707)		Comparison Cohort (n = 177,707)		*p*
	n	%	n	%	n	%	
Age, years (Mean ± SD)	45.86 (15.99)		45.86 (15.99)		45.86 (15.99)		0.989
18–44	181,687	51.12	90,835	51.12	90,852	51.12	0.954
45–64	123,451	34.73	61,703	34.72	61,748	34.75	
≥65	50,276	14.15	25,169	14.16	25,107	14.13	
Gender							
Female	220,030	61.91	110,015	61.91	110,015	61.91	1.000
Male	135,384	38.09	67,692	38.09	67,692	38.09	
CCI							
0	194,006	54.59	81,055	45.61	112,951	63.56	<0.001
1	91,947	25.87	52,584	29.59	39,363	22.15	
2	40,627	11.43	25,544	14.37	15,083	8.49	
≥3	28,834	8.11	18,524	10.42	10,310	5.80	
Diabetes							
No	314,679	88.54	154,345	86.85	160,334	90.22	<0.001
Yes	40,735	11.46	23,362	13.15	17,373	9.78	
Hypertension							
No	279,238	78.57	133,280	75.00	145,958	82.13	<0.001
Yes	76,176	21.43	44,427	25.00	31,749	17.87	
Dyslipidemia							
No	297,582	83.73	142,992	80.47	154,590	86.99	<0.001
Yes	57,832	16.27	34,715	19.53	23,117	13.01	
Atrial fibrillation							
No	347,852	97.87	173,439	97.60	174,413	98.15	<0.001
Yes	7562	2.13	4268	2.40	3294	1.85	
Aspirin							
<28 days	303,329	85.35	146,200	82.27	157,129	88.42	<0.001
≥28 days	52,085	14.65	31,507	17.73	20,578	11.58	
Statin							
<28 days	302,219	85.03	146,471	82.42	155,748	87.64	<0.001
≥28 days	53,195	14.97	31,236	17.58	21,959	12.36	
RAA							
<28 days	279,836	78.74	133,815	75.30	146,021	82.17	<0.001
≥28 days	75,578	21.26	43,892	24.70	31,686	17.83	
Metformin							
<28 days	320,359	90.14	158,390	89.13	161,969	91.14	<0.001
≥28 days	35,055	9.86	19,317	10.87	15,738	8.86	
Level of Urbanization							
Urban	287,041	80.76	144,062	81.07	142,979	80.46	<0.001
Suburban	50,870	14.31	25,100	14.12	25,770	14.50	
Rural	17,503	4.92	8545	4.81	8958	5.04	
Monthly income (NT$)							
0	19,481	5.48	8718	4.91	10,763	6.06	<0.001
1–21,000	141,020	39.68	69,791	39.27	71,229	40.08	
21000–33,300	97,868	27.54	50,614	28.48	47,254	26.59	
≥33,301	97,045	27.30	48,584	27.34	48,461	27.27	

**Table 2:** Risk of Colon Cancer among Sleep Disorders and Comparison Cohort in Study Cohort.

Whole Cohort (n = 355,414)	Sleep Disorders (Total Follow-Up 1,202,268.0 Person-Years)		Comparison Cohort (Total Follow-Up 1,218,949.4 Person-Years)		Adjusted HR ^†^ (95% CI)
	No. of Patients With Cancer	Incidence Rate (per 10^5^ Person-Years) (95% CI)	No. of Patients with Cancer	Incidence Rate (per 10^5^ Person-Years) (95% CI)	
Whole cohort					
Patients	1949	162 (154.9, 169.3)	1415	116 (110.0, 122.1)	1.32 (1.23, 1.42) ***
Age, 18–44 ^a^					
Patients	347	56.3 (50.3, 62.2)	198	31.8 (27.4, 36.2)	1.66 (1.39, 1.98) ***
Age, 45–64 ^b^					
Patients	845	203.7 (189.9, 217.4)	617	146.0 (134.5, 157.6)	1.26 (1.13, 1.40) ***
Age, ≥65 ^c^					
Patients	757	443.6 (412.0, 475.2)	600	344.8 (317.2, 372.4)	1.27 (1.14, 1.42) ***
Female ^d^					
Patients	1071	141.4 (133.0, 149.9)	736	96.1 (89.1, 103.0)	1.38 (1.25, 1.51) ***
Male ^e^					
Patients	878	197.3 (184.3, 210.4)	679	149.9 (138.7, 161.2)	1.27 (1.14, 1.40) ***

^a^ Total follow-up of 616,708.7 person-years for sleep disorders and 622,446.9 for comparison cohort. ^b^ Total follow-up of 414,912.4 person-years for sleep disorders and 422,492.2 for comparison cohort. ^c^ Total follow-up of 170,646.9 person-years for sleep disorders and 174,010.3 for comparison cohort. ^d^ Total follow-up of 757,315.7 person-years for sleep disorders and 766,094.1 for comparison cohort. ^e^ Total follow-up of 444,952.4 person-years for sleep disorders and 452,855.4 for comparison cohort. CI: confidence interval; HR: hazard ratio; ***: *p* < 0.001; ^†^ Main model is adjusted for age, sex, Charlson comorbidity index, diabetes, hypertension, dyslipidemia, AF, level of urbanization, monthly income.

**Table 3:** Sensitivity Analysis of Adjusted HRs of Sleep Disorders and Comparison in Risk of Colon Cancer.

	Comparison Cohort (n = 177,707)	Patients with Sleep Disorders			
		Sleep Apnea (n = 4018)	Insomnia (n = 66,648)	Sleep Disturbance (n = 99,789)	Others (n = 7252)
	Adjusted HR (95% CI)	Adjusted HR (95% CI)	Adjusted HR (95% CI)	Adjusted HR (95% CI)	Adjusted HR (95% CI)
Main model ^†^	1.00	1.17 (0.82, 1.68)	1.42 (1.31, 1.55) ***	1.27 (1.17, 1.38) ***	1.00 (0.77, 1.29)
Additional covariates ^‡^					
Main model + Aspirin	1.00	1.22 (0.85, 1.75)	1.48 (1.36, 1.61) ***	1.31 (1.20, 1.42) ***	1.03 (0.80, 1.33)
Main model + Statin	1.00	1.21 (0.85, 1.73)	1.46 (1.34, 1.59) ***	1.29 (1.19, 1.41) ***	1.02 (0.79, 1.31)
Main model + RAA	1.00	1.25 (0.87, 1.78)	1.48 (1.36, 1.61) ***	1.31 (1.20, 1.42) ***	1.03 (0.80, 1.33)
Main model + Metformin	1.00	1.18 (0.82, 1.68)	1.43 (1.31, 1.55) ***	1.27 (1.17, 1.38) ***	1.00 (0.78, 1.29)
Subgroup effects					
Age, years					
18–44	1.00	1.92 (0.98, 3.77)	1.93 (1.54, 2.43) ***	1.57 (1.29, 1.92) ***	0.91 (0.46, 1.77)
45–64	1.00	1.22 (0.76, 1.96)	1.41 (1.24, 1.60) ***	1.16 (1.02, 1.32) *	0.87 (0.56, 1.36)
≥65	1.00	0.57 (0.21, 1.52)	1.30 (1.14, 1.48) ***	1.28 (1.11, 1.47) ***	1.13 (0.79, 1.60)
Sex					
Female	1.00	1.22 (0.61, 2.45)	1.55 (1.38, 1.73) ***	1.27 (1.13, 1.42) ***	0.90 (0.62, 1.32)
Male	1.00	1.15 (0.76, 1.75)	1.29 (1.13, 1.46) ***	1.27 (1.12, 1.44) ***	1.09 (0.77, 1.53)
CCI					
0	1.00	1.53 (0.84, 2.77)	1.63 (1.42, 1.87) ***	1.41 (1.23, 1.60) ***	0.91 (0.60, 1.38)
1	1.00	1.05 (0.52, 2.12)	1.37 (1.17, 1.60) ***	1.16 (0.99, 1.36)	1.27 (0.83, 1.93)
2	1.00	1.92 (1.04, 3.54) *	1.33 (1.08, 1.64) **	1.10 (0.88, 1.37)	1.19 (0.66, 2.13)
≥3	1.00	0.14 (0.02, 1.02)	1.11 (0.90, 1.37)	1.19 (0.96, 1.47)	0.41 (0.15, 1.10)
Diabetes					
No	1.00	1.42 (0.98, 2.07)	1.43 (1.30, 1.57) ***	1.29 (1.17, 1.41) ***	0.95 (0.71, 1.28)
Yes	1.00	0.43 (0.14, 1.34)	1.34 (1.13, 1.60) ***	1.17 (0.97, 1.41)	1.12 (0.68, 1.86)
Dyslipidemia					
No	1.00	1.05 (0.65, 1.70)	1.44 (1.31, 1.59) ***	1.29 (1.17, 1.42) ***	0.91 (0.67, 1.24)
Yes	1.00	1.30 (0.76, 2.22)	1.34 (1.14, 1.58) ***	1.19 (1.01, 1.42) *	1.25 (0.80, 1.97)
Hypertension					
No	1.00	1.37 (0.86, 2.19)	1.63 (1.46, 1.83) ***	1.31 (1.18, 1.46) ***	0.89 (0.63, 1.26)
Yes	1.00	0.93 (0.54, 1.62)	1.17 (1.03, 1.33) *	1.18 (1.03, 1.35) *	1.13 (0.78, 1.64)
Atrial fibrillation					
No	1.00	1.22 (0.85, 1.74)	1.44 (1.32, 1.57) ***	1.26 (1.16, 1.38) ***	0.99 (0.76, 1.29)
Yes	1.00	-			
Aspirin					
<28 days	1.00	1.51 (1.02, 2.23) *	1.71(1.55, 1.89) ***	1.44(1.30, 1.58) ***	1.24(0.94, 1.65)
≥28 days	1.00	0.56 (0.23, 1.36)	0.96(0.81, 1.13)	0.93(0.78, 1.11)	0.53(0.29, 0.97) *
Statin					
<28 days	1.00	1.04 (0.67, 1.62)	1.53(1.41, 1.71) ***	1.36(1.24, 1.49) ***	1.03(0.77, 1.37)
≥28 days	1.00	1.66 (0.90, 3.05)	1.11(0.91, 1.35)	1.01(0.82, 1.24)	0.95(0.55, 1.67)
RAA					
<28 days	1.00	1.38 (0.89, 2.16)	1.71(1.54, 1.90) ***	1.44(1.30, 1.59) ***	1.04(0.75, 1.43)
≥28 days	1.00	0.99 (0.54, 1.80)	1.11(0.96, 1.28)	1.03(0.88, 1.20)	0.98(0.64, 1.49)
Metformin					
<28 days	1.00	1.14 (0.76, 1.69)	1.44 (1.31, 1.58) ***	1.29 (1.18, 1.41) ***	0.95 (0.72, 1.27)
≥28 days	1.00	1.39 (0.61, 3.15)	1.34 (1.08, 1.67) **	1.15 (0.91, 1.46)	1.25 (0.69, 2.23)

ICD-9-CM: sleep apnea: 780.51, 780.53, and 780.57; insomnia: 780.52; sleep disturbance: 780.5 (excluded 780.51, 780.53, and 780.57); others: 307.4, 780.50, 780.54–780.56, 780.58–780.59. *: *p* < 0.05 **: *p* < 0.01 ***: *p* < 0.001; HR: hazard ratio; CCI Index: Charlson Comorbidity Index. ^†^ Main model is adjusted for age, sex, Charlson comorbidity index, diabetes, hypertension, dyslipidemia, AF, level of urbanization, and monthly income. ^‡^ The models were adjusted for covariates in the main model as well as each additional listed covariate.

**Table 4:** Risk of Colon Cancer among Sleep apnea, Insomnia, and Comparison Cohort in Study Cohort.

	Comparison Cohort (n = 70,666)	Sleep Apnea Only (n = 3256)	Insomnia Only (n = 64,996)	Sleep Apnea+ Insomnia (n = 2414)
	Adjusted HR (95% CI)	Adjusted HR (95% CI)	Adjusted HR (95% CI)	Adjusted HR (95% CI)
Main model ^†^	1.00	1.08 (0.69, 1.69)	1.45 (1.31, 1.61) ***	1.66 (1.21, 2.29) **
Subgroup effects				
Age, years				
18–44	1.00	1.83 (0.78, 4.27)	2.19 (1.60, 3.00) ***	2.57 (1.17, 5.67) *
45–64	1.00	1.17 (0.65, 2.09)	1.39 (1.18, 1.63) ***	1.57 (1.00, 2.49)
≥65	1.00	0.45 (0.11, 1.82)	1.36 (1.17, 1.58) ***	1.51 (0.86, 2.63)
Sex				
Female	1.00	1.06 (0.39, 2.83)	1.59 (1.38, 1.83) ***	1.93 (1.20, 3.11) **
Male	1.00	1.05 (0.63, 1.75)	1.31 (1.13, 1.53) ***	1.46 (0.95, 2.26)

CI: confidence interval; HR: hazard ratio; *: *p* < 0.05 **: *p* < 0.01 ***: *p* < 0.001; ^†^ Main model is adjusted for age, sex, Charlson comorbidity index, diabetes, hypertension, dyslipidemia, AF, level of urbanization, and monthly income.

## Data Availability

The data supporting the findings of this research were sourced from NHIRD in Taiwan. Owing to the legal restrictions imposed by the Government of Taiwan related to the Personal Information Protection Act, the database cannot be made publicly available.
